# Supplementary material for: Neutrophil polarization by IL-27 as a therapeutic target for intracerebral hemorrhage
Source: Nat Commun. 2017 Sep 19;8:602. doi: 10.1038/s41467-017-00770-7 (PMC5605643; doi:10.1038/s41467-017-00770-7)
Supplement: Supplementary file 1 — Supplementary Information [file 41467_2017_770_MOESM1_ESM.pdf]

Description of Supplementary Files

File name: Supplementary Information
Description: Supplementary figures, supplementary tables

File name: Peer Review File

Supplementary Figure 1. Gating Strategy. The FACS gating strategy of lactoferrin intensity in blood PMNs presented in **Figure 3e** (a) and the bone marrow expression of LTF in **Figure 5c** (b).

A.

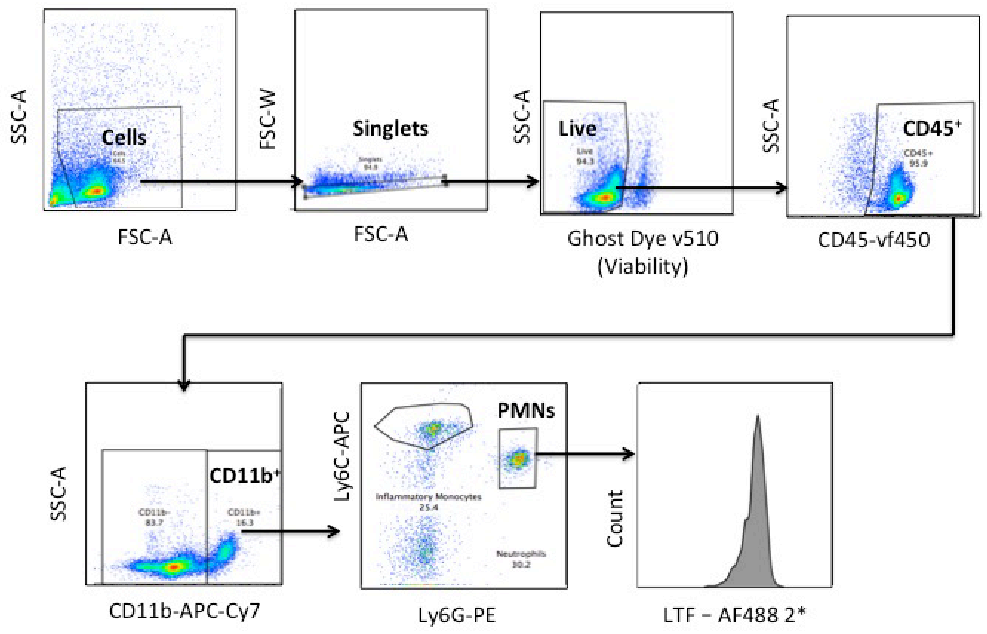

B.

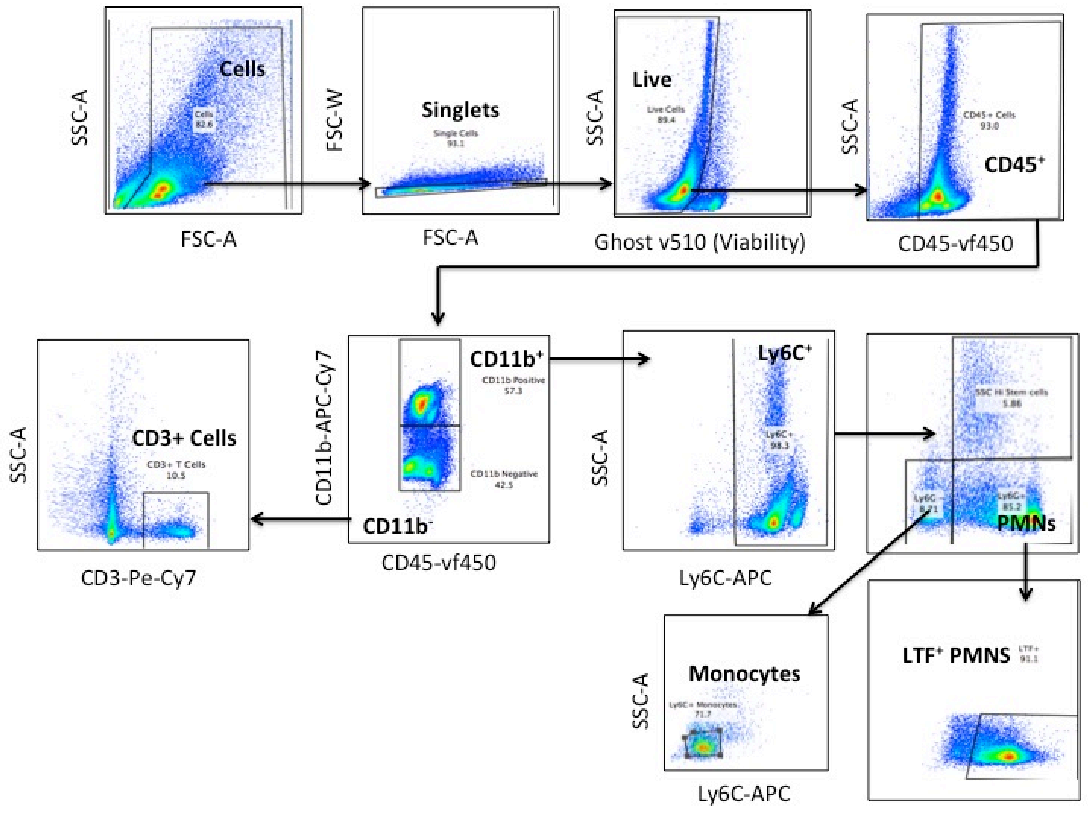

Supplementary Figure 2: Western Blots of Fig. 2g

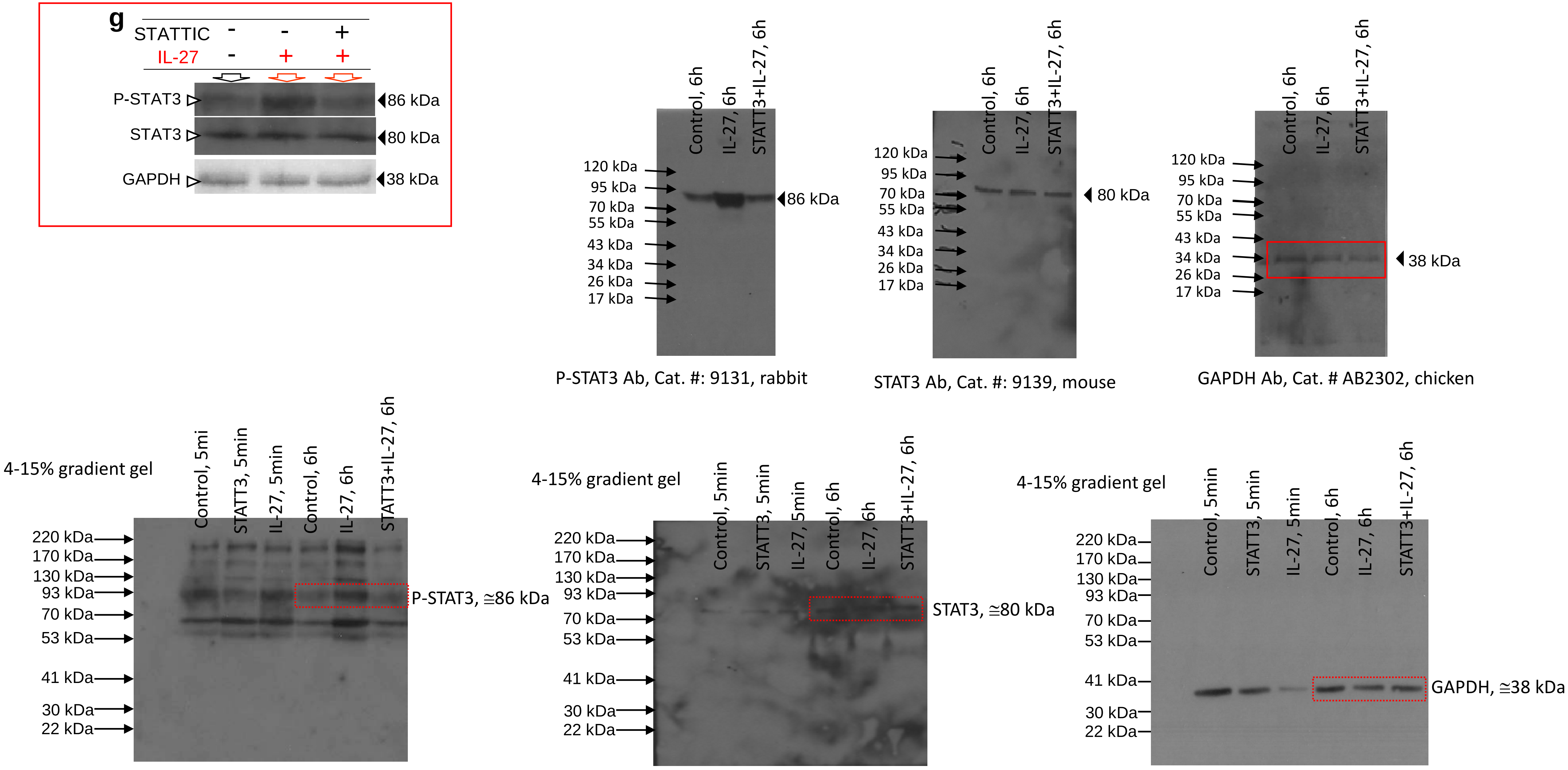

Supplementary Figure 3: Western Blots of Fig. 5g

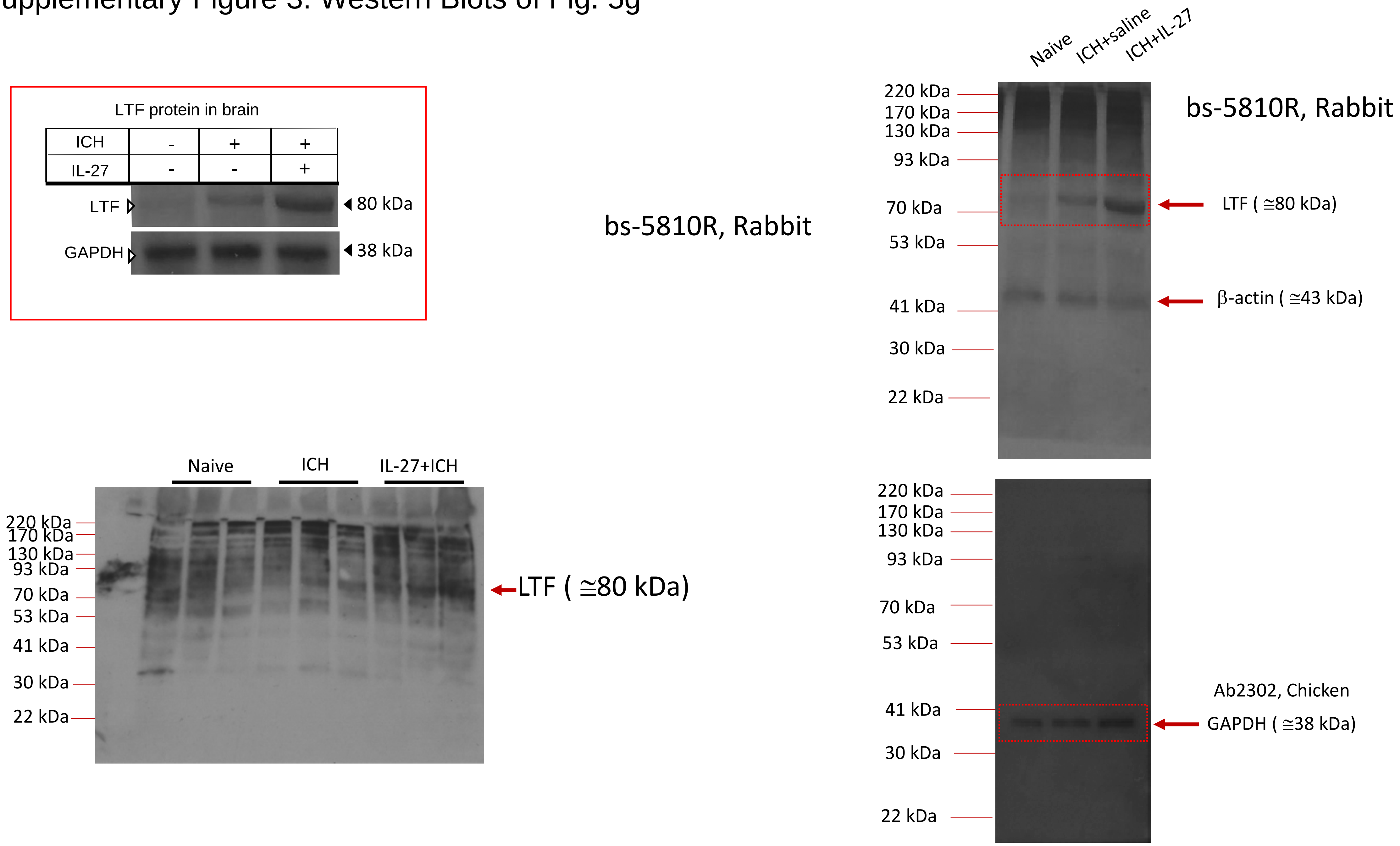

Supplementary Table 1 - PCR Primers

Gene Name	Species	Primer Sequence		Location of Primer	Gene ID	Product size (bp)
IL27 p28	rat	F	tgaggttcagggctatgtcc	165-	XM_344962.4	353
		R	cctcctcctcgtcctcttct	-517		
EBI3	rat	F	tcagggttcccgtcactatc	106-	NM_001109421	342
		R	aatgaaggacatggctctgg	-447		
IL-27Rα	rat	F	ctatggctggcctgaaactc	1753-	NM_001105943	358
		B	aacgtgtcttccgaatcacc	-2110		
gp130	rat	F	gcaagaagatggcactgtga	1426-	NM_001008725	375
		R	gtgtatgctgccatgtggac	-1800		
GAPDH	rat	F	agacagccgcatcttcttgt	28-	NM_017008	323
		R	tactcagcaccagcatcacc	-350		
NF-L	rat	F	aaaggtgcacgaggaagaga	743-	NM_031783.1	362
		R	tgtctgcattctgcttgtcc	-1104		
GFAP	rat	F	ggatgtgatgtgaggtgtgc	1306-	NM_017009	433
		R	cctcgggatcttttcctttc	-1738		
MBP	rat	F	acttggccacagcaagtacc	84-	M25889	312
		R	tgtctcttcctccccagcta	-395		
CD68	rat	F	aatgtgtccttcccacaagc	712-	NM_001031638	328
		R	agaggggctggtaggttgat	-1039		
IL-27 p28	mouse	F	gaggttcagggctatgtcca	166-	NM_145636.1	374
		R	aggggcagcttcttttcttc	-539		
IL-27Rα	mouse	F	acccaaatgaagccagacac	396-	NM_016671	414
		R	cacacaaggtcttgggtcct	-809		
GP130	mouse	F	tccgtgcaagtgttctcaag	2594-	NM_010560	459
		R	tcaggagccagtccttcact	-3052		
LTF	mouse	F	cgaagcacgaatgacaaaga	860-	NM_008522	306
		R	atcacacttgcgcttctcct	-1165		
Hp	mouse	F	ctctacgtggggaaaaacca	538-	NM_017370	353
		R	cagaaggtgtgctcgttcaa	-890		
NOX2	mouse	F	actgcggagagtttggaaga	757-	FJ168469	427
		R	gcttatcacagccacaagca	-1183		
ELANE	mouse	F	ggctttgacccatcacaact	342-	NM_015779	440
		R	aggtggtcattatggcttcg	-748		
MPO	mouse	F	atgcaccaggaacaacatca	1170-	NM_010824	448
		R	caacaccaagggcaggtagt	-1617		
MMP9	mouse	F	cgtcgtgatccccacttact	651-	NM013599	433
		R	agagtactgcttgcccagga	-1083		
iNOS	mouse	F	gtggtgacaagcacatttgg	1928-	NM010927	487
		R	ggctggacttttcactctgc	-2414		
GAPDH	mouse	F	tgttcctacccccaatgtgt	754-	NM_001001303	396
		R	tgtgagggagatgctcagtg	-1149		

Supplementary Table 2 - Antibody list (excluding Flow)

Name of Ab	Cat. #	Source of Ab	Company	Application	Usage
LTF	L3262	rabbit	Sigma	IF and Flow	rat/mouse
LTF	07-687	rabbit	Millipore	IF and WB	mouse
LTF	bs-5810R	rabbit	Bioss	WB	rat
IL-27p28	ab62501	rabbit	Abcam	IF	mouse
STAT3	9139	mouse	Cell Signaling	WB	rat
p-STAT3	9131	rabbit	Cell Signaling	WB	rat
GAPDH	AB2302	chicken	Millipore	WB	rat/mouse
MAP2	M4403	mouse	Sigma	IF	rat
GFAP	G9269	rabbit	Sigma	IF	rat
Neutrophil (ELANE)	ab53457	rat	Abcam	IF	mouse
MPO	A0398	rabbit	DAKO	IF	rat
RP-1	550000	mouse	BD Pharmingen	IF	rat
Iba-1	019-19741	rabbit	Wako	IF	rat
NeuN	MAB377	mouse	Chemicon	IF	rat
CD68	HM1070	rat	HycultBiotech	IF	mouse
Ly-6G	BE0075	rat	BioXCell	Neutralizing	mouse
rat IgG 2a	BE0089	rat	BioXCell	control for BE0075	mouse
IL-27 p28	516912	mouse	Biolegend	Neutralizing	mouse
Rat IgG 1	400427	rat	Biolegend	Control for 516912 for exp. Fig 3B	mouse
mouse IgG2a	400264	mouse	Biolegend	Control for 516912 for exp. Fig 3A	mouse
Rabbit IgG isotype control	I-1000	rabbit	Vector	Control for L3262	mouse

Supplementary Table 3 - Flow Cytometry Staining Reagents & Antibodies

Antibody	Fluorophore	Origin	Target	Dilution	Clone	Source
Viability and Blocking						
Ghost Dye	Violet 510	N/A	Amine Reactive Viability Dye	1:1000	N/A	Tonbo Biosciences
Anti-Mouse CD16/CD32 (Purified)	N/A	Rat IgG2b	FC Receptor Blockade	1:100	2.4G2	Tonbo Biosciences
Extracellular Antibodies						
Anti-Mouse CD45	VioletFluor450	Rat IgG2b	Leukocyte Common Antigen	1:40	30-F11	Tonbo Biosciences
Anti-Human/Mouse CD11b	APC-Cyanine7	Rat IgG2b	Macrophages, Granulocytes, NK cells	1:80	M1/70	Tonbo Biosciences
Anti-Mouse Ly6G	PE	Rat IgG2a	Neutrophils	1:70	1A8	Biolegend
Anti-Mouse Ly6C	APC	Rat IgG2c	Inflammatory monocytes, neutrophils, T cell sub-sets	1:80	HK1.4	Biolegend
Anti-Mouse CD3	PE-Cyanine 7	Rat IgG2b	T Cells	1:80	17A2	Biolegend
Intracellular Antibodies						
Rabbit Anti-Lactoferrin	None	Rabbit	Lactoferrin (LTF)	1:100	L3262	Sigma Aldrich
Rabbit IgG	None	Rabbit	Isotype Control (LTF)	1:100	I-1000	Vector
Goat anti-rabbit IgG	AlexaFluor488	goat	Secondary Antibody	1:1000	A11029	Invitrogen
